# Understanding cancer as a systemic disease through comprehension of neural stemness as the core property of cancer cell and the basic rules it dictates

**DOI:** 10.3389/fcell.2026.1843646

**Published:** 2026-08-05

**Authors:** Ying Cao

**Affiliations:** 1 The MOE Key Laboratory of Model Animals for Disease Study, Model Animal Research Center, Medical School of Nanjing University, Nanjing, China; 2 Jiangsu Key Laboratory of Molecular Medicine, Medical School of Nanjing University, Nanjing, China; 3 Shenzhen Research Institute of Nanjing University, Shenzhen, China

**Keywords:** cancer cell, differentiation, neural default model of embryonic pluripotent cells, neural induction, neural stemness, pluripotency, tumorigenesis, tumorigenicity

## Abstract

Cancer is a systemic disease with multilayered complexity. Some theories/hypotheses have been proposed to explain cancer. They are successful in explaining certain aspects of cancer, but meet serious challenges in other aspects. Inappropriate understanding of cancer cell and cancer complexity also hinders the development of more effective strategies of cancer therapy. My previous studies demonstrated that the core property of cancer (tumorigenic) cells is neural stemness. The finding led to the subsequent discovery about the central role of neural stemness during tumorigenesis and a paradigm that can hopefully explain systemic complexity of cancer as a whole. In the review, I summarize the evidence from research of evolutionary, developmental and cancer biology supporting that neural stemness, representing the general stemness predestined by its evolutionary advantage, determines both pluripotency and tumorigenicity, the key cellular properties underlying developmental and cancer biology, respectively. I made detailed discussions about the central role of neural stemness in understanding the core property and phenotypic traits of cancer cell and in understanding phenotypic heterogeneity in cancer. These pieces of evidence and discussions reveal that cancer is the manifestation of the power of basic rules dictating both embryogenesis and tumorigenesis. Briefly, acquiring neural stemness, hence a pluripotent state in ectodermal cells during embryogenesis, leads to neural development and body axis formation, i.e., embryonic neural induction, and ectopic neural induction causes a conjoined twin (secondary body axis); whereas acquirement of neural stemness, hence a pluripotent state in cells of a postnatal animal/human, results in a degenerated conjoined twin-like structure, i.e., a tumor. Tumors as conjoined twin-like structures formed in postnatal animals/humans helps understand the complex crosstalks within tumors and crosstalks between tumors and hosts. Moreover, neural stemness being the core property of cancer cell should account for the inverse correlation between cancer and neurodegeneration, and the neurodegeneration effect in patients after cancer therapies. Due to the central role of neural stemness in contributing to tumorigenesis, novel strategies of cancer therapy can be developed by targeting neural stemness using the principle of pluripotent cell differentiation. In addition, some essential issues worth considering in cancer research are also discussed.

## 1 Introduction

Cancer is recognized as a systemic disease because of its multi-dimensional complexity (Swanton et al., 2024). Cancer research has revealed that almost all aspects of biological research are accountable for cancer, such as from the finest molecular details to evolutionary ecosystem (Bhattacharya et al., 2025; Merlo et al., 2006), from embryonic development to aging (Swanton et al., 2024; Rubin, 1985; Stanger and Wahl, 2024), from genetic heterogeneity (Turajlic et al., 2019) to phenotypic heterogeneity (Meacham and Morrison, 2013), and to the heterogeneity of a particular biological process involved in cancer, for example, metabolic heterogeneity (Tong et al., 2020). Molecular studies on cancer cells and different elements in tumor microenvironment (TME), including different types of cells and even microbiome, revealed that nearly every gene can be associated with cancer (de Magalhães, 2022). Such a scenario of systemic complexity means that dealing with cancer is almost dealing with the whole animal world. In the past time, cancer has been understood primarily based on reductionist cell biology, and molecular mechanism-driven cancer research has produced extraordinary discoveries that help understanding cancer cells and their local microenvironment. But frustratingly, very few innovative new therapeutic strategies that are broadly beneficial across different cancers and significantly prolong overall survival have been developed, and therapy resistance is always the insurmountable obstacle (Swanton et al., 2024). Dilemma of this reality prompts to rethink of cancer more inclusively to reflect the multidimensional complexity of disease mechanisms, instead of focusing only on the reductionist view of shared hallmarks of cancer. Novel and more efficient therapeutic strategies should be developed based on the systemic complexity of cancer (Swanton et al., 2024; Bhattacharya et al., 2025). Cancer is a disease with multidimensional complexity. If looking at the complex elements, e.g., cell types, interactions, etc., in the scenario of tumor ecosystem, it raises the question what is the causality in the ecosystem and what are the causal and supporting elements. The answer to the question will improve the understanding cancer as a systemic disease and help to identify the key factor to consider for developing novel therapeutic strategies. Cancer is a complex derivative from normal cells and contains almost all elements in animals. Therefore, tumorigenesis should also follow the rules operating in the animal kingdom. My studies identified neural stemness, which represents the general stemness, as the core property of cancer (tumorigenic) cells, which led to the subsequent finding of general rules that governs both tumorigenesis and embryogenesis. These rules explain complexity of cancer as the derivative from neural stemness. In the review, I will discuss the role of neural stemness as the cornerstone in understanding tumorigenesis. Moreover, I will also discuss misconceptions and ambiguities that may complicate the research and understanding of cancer.

## 2 Neural stemness represents the general stemness

By intuition, neural stemness is recognized as a type of tissue stemness. But further analysis and integration of information from evolutionary and developmental biology research suggest otherwise.

### 2.1 Pluripotency of neural stem cells (NSCs)

Embryonic pluripotent cells, such as amphibian blastula ectodermal cells and mammalian embryonic stem cells (ESCs), are considered as the basis for differentiation because of their ability of differentiation into all types of cells during normal embryogenesis and in experimental conditions. Neural stem/progenitor cells and neural crest cells (NCCs) appear later than embryonic pluripotent cells during embryonic development and their obvious contribution to embryonic development is formation of the nervous system. Therefore, neural stemness, referring to the collective property of primitive NSCs, NCCs, adult NSCs and neural progenitor cells, was naturally considered as a type of tissue stemness. This view has considered simply the obvious properties of embryonic pluripotent cells and neural stemness. The hidden relationship between the stemness of embryonic pluripotent cells and neural stemness has been overlooked. During gastrulation of amphibian embryogenesis, neuroectoderm is formed via a process called “neural induction”, in which the ectoderm loses its epidermal fate and acquires neuroectodermal fate in response to inhibition of TGFβ signaling (Cao, 2023). Neuroectoderm gives rise to neural plate, which contributes to formation of not only the central nervous system, but non-neural cells as well during later developmental stage. In the most posterior region of elongating embryos, neuromesodermal progenitors, which are presumably originated from anterior neural plate, generate both spinal cord and paraxial mesoderm (Henrique et al., 2015; Sambasivan and Steventon, 2021). Locating between neural plate and epidermal ectoderm, neural crest is induced by interactions between neural plate and adjacent tissues (Selleck and Bronner-Fraser, 1995; Knecht and Bronner-Fraser, 2002; Pla and Monsoro-Burq, 2018). It is well characterized that NCCs are pluripotent because of they are not just the precursors of the peripheral nervous system. They also contribute to a variety of non-neural tissues, such as melanocytes, skeletal and connective tissues, and medulla cells of the adrenal gland (Le Douarin and Dupin, 2016). Meanwhile, pluripotent property of NCCs is supported by a pluripotency-like molecular program in these cells (Pajanoja et al., 2023; Zalc et al., 2021). Developmental relationship between neural plate and neural crest means that pluripotency of NCCs is manifestation of the property of neural plate cells, i.e., the primitive NSCs. Pluripotency of NSCs is not obvious during embryogenesis, but was experimentally verified (Cao, 2022; Clarke et al., 2000; Tropepe et al., 2001; Xu et al., 2021; Zhang et al., 2022). NSCs also have pluripotency-like molecular program. The four original reprogramming factors, Sox2, c-Myc, Oct4 and Klf4, subsequent alternative reprogramming factors, and reprogramming co-regulators, are all enriched in neural precursor/progenitor cells during vertebrate embryonic development (Cao, 2022).

### 2.2 The “neural default state” of embryonic pluripotent cells

Pluripotency of NSCs can be traced back to the “neural default model” of embryonic pluripotent cells during embryogenesis (Muñoz-Sanjuán and Brivanlou, 2002). Ectoderm is the germ layer that gives rise to both epidermis and nervous system, and other tissues/organs are mostly derived from either endoderm or mesoderm. How the neural tissue in an early embryo is induced to form had been a major topic of research in developmental biology. Spemann and Mangold (1924) demonstrated that dorsal blastopore lip, or the Spemann-Mangold organizer, of an early newt gastrula embryo was able to induce neural plate in ectoderm when transplanted into the ventral side of a host embryo, while the dorsal lip itself developed into mesodermal notochord. After a tortuous process in exploration of the mechanisms underlying neural inducing activity by the organizer, it was elucidated that absence, but not presence, of an extracellular signal is prerequisite for neural fate decision, suggesting that neural fate might be the default fate of ectoderm (Godsave and Slack, 1989; Grunz and Tacke, 1989; Sato and Sargent, 1989). In agreement, the organizer is the rich source of secreted factors antagonizing BMP4, a TGFβ ligand that transduces epidermis-inducing and anti-neural signaling in ectoderm (De Robertis, 2006; De Robertis and Kuroda, 2004; Harland, 2000). It was concluded that neural fate is achieved by default and epidermal fate is induced during ectodermal cell fate decision, i.e., the “neural default model” of ectoderm (Muñoz-Sanjuán and Brivanlou, 2002). Amphibian blastula ectodermal cells were further validated to be the equivalent of mammalian ESCs, which also follow the “neural default model.” They adopt neural fate and turn into primitive NSCs in absence of extracellular inducers (Tropepe et al., 2001; Smukler et al., 2006).

### 2.3 Unicellular origin of neural stemness

The neural default state of pluripotency is rooted in the evolutionary advantage of neural genes and neural stemness. During evolution, origin of ectoderm is the earliest, followed sequentially by endoderm and mesoderm. Comparison of evolutionary origin of neural and non-neural genes in ectoderm showed that one peak of emergence of neural genes is already present in the time point representing the last common ancestors of eukaryotes and the other is at the time of emergence of eumetazoa (Dom et al., 2007), indicating that neural genes have an earlier evolutionary origin and hence, play a critical role in multicellularity. Detailed analysis on more than 5,000 neural genes in vertebrates demonstrated that most of these genes can be traced back to *Monosiga brevicollis*, *Amphimedon queenslandica* and *Trichoplax adhaerens*, which are the closest species representing transition from unicellularity to multicellularity, and share a last common unicellular ancestor in more than 600 million years ago. *M. brevicollis* represents the closest unicellular relatives of metazoans, *Amphimedon queenslandica* is the oldest surviving metazoan and an evolutionary intermediary between unicellular choanoflagellate protists and eumetazoans, and *T. adhaerens* is the basal species of eumetazoan. Therefore, most ancestral neural genes had emerged during the transition from unicellularity to multicellularity. Importantly, more than 60% of genes in *M. brevicollis* that are homologous to vertebrate genes are ancestral neural genes, suggesting that the last common unicellular ancestor was biased towards a neural state (Xu et al., 2021). The notion is further supported by identification of neurosecretory apparatus in *M. brevicollis* and unicellular origin of neurosecretory cell-cell signalling (Burkhardt et al., 2011; Göhde et al., 2021).

This means that neural biased state is the ground state or the starting cellular state for the transition from unicellularity to multicellularity during evolution. In addition, genes coding for the components of machineries required for basic cellular physiological functions and developmental programs are mostly enriched in embryonic neural cells, such as cell cycle, ribosome, spliceosome, proteasome, epigenetic modification, reprogramming, DNA damage and repair. They work concerted together to define neural stem/embryonic neural cells as a highly proliferative and pluripotent state. These basic machineries are common to eukaryotes, hence, have a unicellular origin. Embryonic neural cells represent the direct descendant cells of the unicellular ancestors (Cao, 2022; Xu et al., 2021; Chen et al., 2021).

Emergence of TGFβ signaling during evolution also suggests that neural state represents the ground state of differentiation. TGFβ signaling is required for inhibition of neural differentiation and promotion of non-neural differentiation of ESCs or during germ layer differentiation (Itoh et al., 2014; Meyers and Kessler, 2017; Ozair et al., 2013). Meanwhile, BMP4, a TGFβ family member, is also required for maintenance of ESC pluripotency, because ESCs adopting a neural fate in the absence of BMP4 signaling has been considered as a differentiation effect (Malaguti et al., 2013; Ying et al., 2003). As a prime signaling promoting non-neural differentiation during embryogenesis, emergence of TGFβ pathway coincided with the onset of multicellularity during evolution, suggesting its role in cell type diversification. The pathway is not present in *M. brevicollis*, but present in *A. queenslandica* (Adamska et al., 2007; Huminiecki et al., 2009; King et al., 2008; Srivastava et al., 2010). Moreover, study on pluripotent cells in *A. queenslandica* also confirmed that pluripotency has a unicellular origin (Sogabe et al., 2019). Therefore, evolutionary studies indicate that neural-biased state of the last common unicellular ancestors represents the ground state of pluripotency (Cao, 2022). Adoption of neural fate of ESCs in the absence of TGFβ signaling should not be interpreted as a differentiation effect, but rather a reversal effect to the most initial state of pluripotency. Evolutionary advantage of neural genes and neural stemness explains why the default fate of embryonic pluripotent cells is neural. TGFβ signaling has been considered to maintain pluripotency. But paradoxically, its inhibition improves reprogramming to generate pluripotent state (Woltjen and Stanford, 2009). The paradox can be resolved if the evolutionary origin of pluripotency and the relationship between ESCs and primitive NSCs are considered. The induced pluripotent stem cells (iPSCs) are equivalent to primitive NSCs rather than ESCs.

**FIGURE 1:**
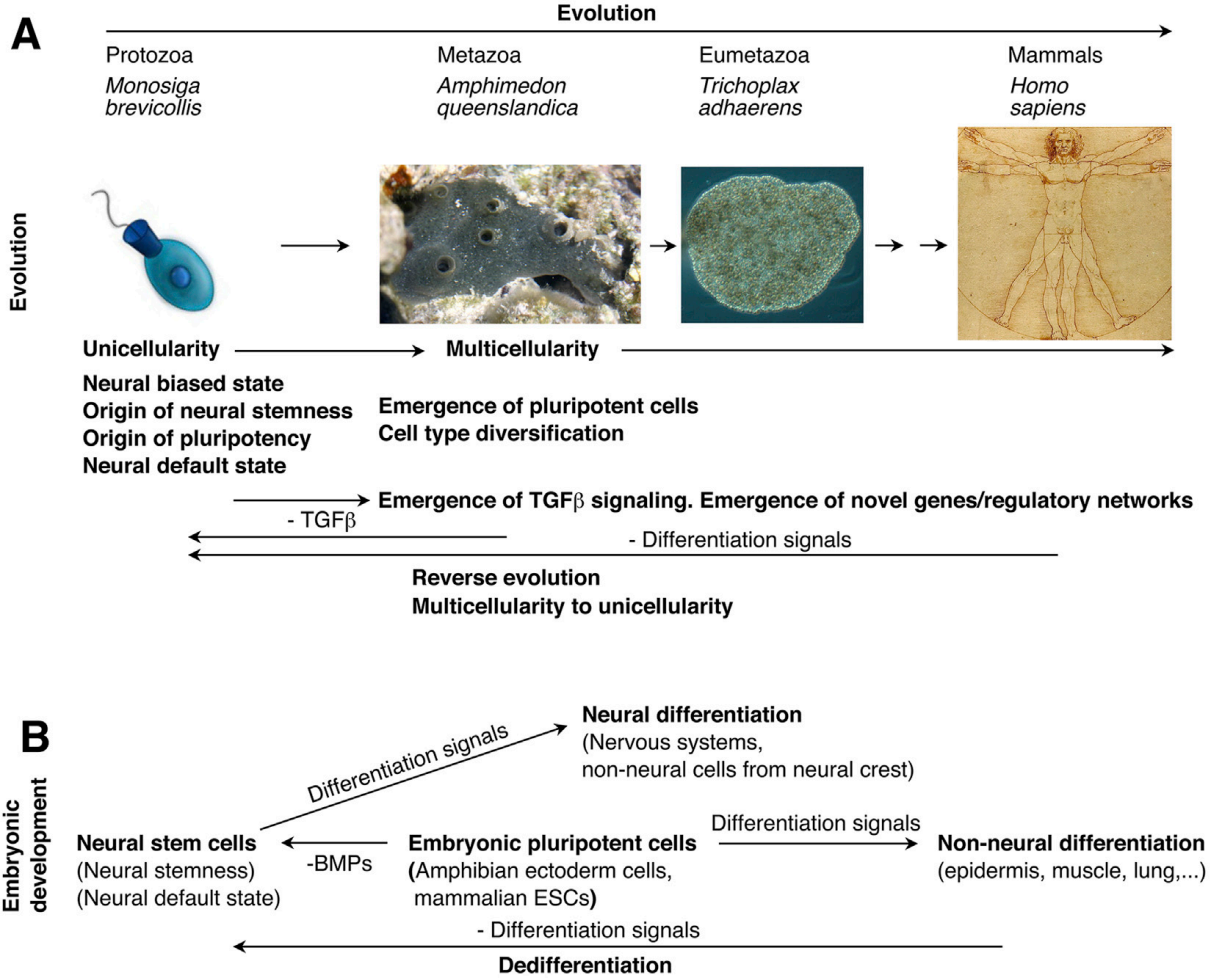
Evolutionary and developmental basis for neural stemness as the general stemness. **(A)** Neural-biased state of the last common unicellular ancestors indicating unicellular origin of neural stemness and pluripotency, which serves as the initial state from unicellularity to multicellularity during animal evolution. Emergence of TGFβ signaling in the basal species of metazoan and other new genes and regulatory networks during evolution promotes cell type diversification in animals. **(B)** The “neural default model” of embryonic pluripotent cells. Embryonic pluripotent cells are capable of neural and non-neural differentiation. For neural differentiation, BMP signaling must be inhibited, e.g., by secreted signals from the Spemann-Mangold organizer, thereby embryonic pluripotent cells turning into primitive neural stem cells, a process determined by evolution as depicted in **(A)**. Loss of BMP signaling in embryonic pluripotent cells and loss of differentiation signals in differentiated cells, i.e., dedifferentiation, reflects an effect of reverse evolution.

In addition to evolutionary advantage, neural genes are characteristic of over-representation of long genes with more exons and introns, as compared with non-neural genes. Neural genes are twice as long as non-neural genes, and have four more exons/introns than non-neural genes in average (Cao, 2022; Xu et al., 2021; Gabel et al., 2015; Zylka et al., 2015). Longer genes with more exon/introns facilitate binding of different regulators or forming secondary structures, and hence, can serve as more flexible scaffolds for diverse regulatory signals during differentiation. More exon/intron compositions mean these genes can generate more splice variants via splicing. Accordingly, components of the machinery of alternative splicing, a mechanism contributing to phenotypic novelty during evolution, and to cell differentiation, lineage determination and organogenesis during development (Baralle and Giudice, 2017; Bush et al., 2017), are enriched in embryonic neural cells (Cao, 2022). By contrast, shorter genes should have no such advantages. Enrichment of longer genes makes neural stemness a more flexible scaffold and an appropriate initial state for cell differentiation. By integrating the evidence from studies on evolution, cellular properties and regulatory networks, and intrinsic association between ESCs and NSCs, it can be concluded that neural stemness represents general stemness, i.e., the ground state of pluripotency. Neural induction is in fact the process of returning to the neural ground state of embryonic pluripotent cells. Unicellular origin of neural stemness and pluripotent state, and neural default state of embryonic pluripotent cells is summarized in Figure 1.

## 3 Neural stemness as the core property of cancer cell

Cancer cell was ever the primary focus of cancer research before 1980s because mutations in oncogenes and tumor suppressor genes are seemingly sufficient to determine cancer initiation and progression. But this cancer cell-intrinsic view has met difficulties in explaining the mechanisms that govern cancer metastasis. Studies on crosstalks in TME and interactions between tumor and host tissues might provide reasonable explanations, leading to a shift of focus on cancer cell to tumor environment (Garner and de Visser, 2020; Maman and Witz, 2018; Vogelstein and Kinzler, 1993). Cell and molecular biology research has revealed a number of phenotypic traits of cancer cell that are usually supposed to be not manifested by normal cells, including fast proliferation, invasion/migration, stemness, evasion of cell death and immune destruction, therapy resistance, dysregulated epigenetics and metabolism. Development of novel therapeutic strategies based on molecular mechanisms that regulate these phenotypic traits has achieved very limited effects. The frustrating situation also incites researchers to consider more comprehensively the complexity of cancer instead of cancer cells themselves (Swanton et al., 2024). It is believed that phenotypic traits of cancer cell, e.g., metastasis, are the consequence of acquirement of “mesenchymal state” via process called “epithelial-mesenchymal transition (EMT)” during tumorigenesis. However, after more than 50 years of extensive EMT study and publication of more than 50K EMT papers, nothing is clear about the basics of EMT, including epithelial and mesenchymal states, EMT-specific markers, clear evidence of EMT, and even basic rationale of EMT, rendering EMT a scientifically groundless and meaningless concept (Cao, 2024; Yang et al., 2020). Understanding cancer cell with a poorly defined “concept” is an obvious violation of scientific reasoning. This raises a question how much cancer cell has been really understood. Major cancer promoting factors are best investigated for their roles in regulating of phenotypic traits of cancer cell. It is common to see that one factor is able to regulate different phenotypic traits of cancer cells. For example, EZH2 plays oncogenic role in different types of cancers, promotes cancer cell stemness (Balinth et al., 2022), proliferation (Bryant et al., 2007), metastasis (Zingg et al., 2015), chemoresistance (Crea et al., 2012; Ougolkov et al., 2008), metabolic dysregulation (Ahmad et al., 2017), immune evasion (Kim et al., 2020; Zhou et al., 2020), etc. c-Myc regulates almost all phenotypic traits, including immune evasion, of cancer cells (Dhanasekaran et al., 2022; Fatma et al., 2022; Llombart and Mansour, 2022). These mean that different phenotypic traits of cancer cells are not independent from each other, but rather, intrinsically connected. Understanding how these traits are intrinsically connected also needs better understanding of the property of cancer cell.

### 3.1 Cancer (tumorigenic) cells are characteristic of neural stem/embryonic neural cells

**FIGURE 2:**
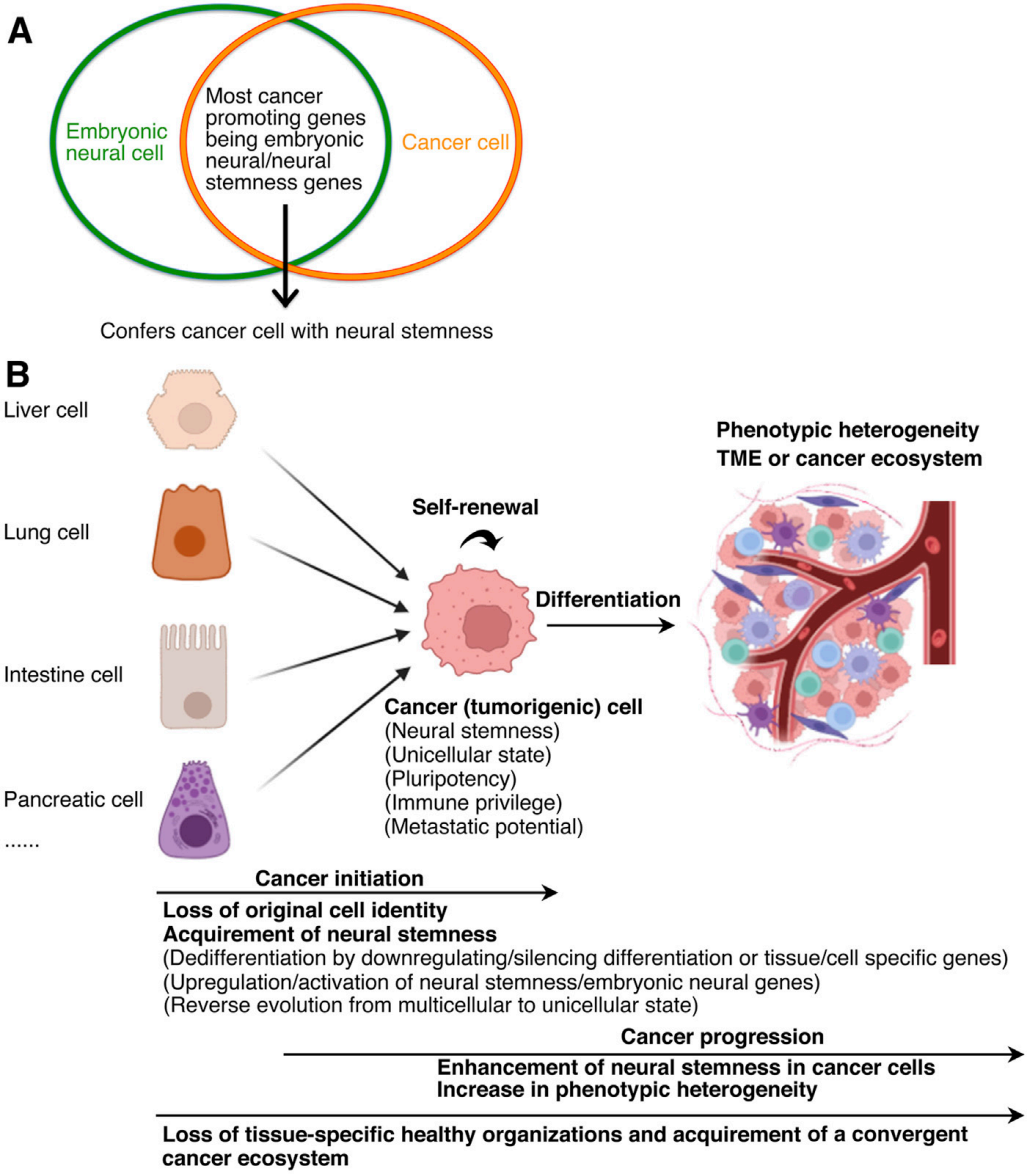
Neural stemness as the core property of cancer cell. **(A)** Most (if not all) cancer promoting genes are specifically expressed or enriched in embryonic neural cells, which confers cancer cell with neural stemness. **(B)** Tumorigenesis represents a process of progressive loss of original cell identity and acquirement of neural stemness. This is a process of reverse evolution from differentiated state to the unicellular state. Acquirement of neural stemness means acquiring pluripotent differentiation potential in cancer cell, which is capable of multilineage differentiation, leading to different types of cells that contribute at least partially to formation of tumor microenvironment (TME) or cancer ecosystem. Acquirement of neural stemness means acquiring immune privilege in cancer cell, which helps cancer cells to evade from immune killing. Acquirement of neural stemness also means acquiring metastatic potential. During cancer progression, there is a tendency of enhancement in neural stemness of cancer cells, hence the increase in differentiation potential or cell plasticity, and consequently, leading to increase in phenotypic heterogeneity. Regardless of the type of tissue of cancer origin, healthy organization of the tissue is disrupted. Meanwhile, neural stemness as the core property of cancer cells gives rise to convergence of cancer ecosystem formation. In addition, tumorigenesis as a process of loss of original cell identity and acquirement of neural stemness, i.e., convergence from multicellular state to a unicellular state, also explains why cancer-driving mutations usually exhibit tissue specificity, because whether or how they can drive cancer depends on whether they can confer cells with neural stemness, a process depending on cellular context. [**(B)** was created partially with Biorender.com].

Cancer cells are immature cells, and differentiation is expected to suppress malignancy of cancer cells. In an initial study, we tried to find out whether it was possible to drive terminal differentiation of different types of cancer cells using a same approach. Instead of using a technical strategy for screening some common molecules that might be involved in differentiation/dedifferentiation of cancer cells, we set up a few limiting rules, which were inferred from characteristics of cancer cells, to narrow down the number of candidate factors (Zhang et al., 2017). These restrictions led us to focusing on HDAC1, HDAC3, EZH2, LSD1 and DNMT1, the best-known epigenetic modification factors that are pan-cancer promoting proteins (Zhang et al., 2017). Interestingly, combined inhibition of these oncoproteins led to post-mitotic neuronal-like differentiation in cells of different types of cancer, including hepatocellular carcinoma, prostate cancer, breast cancer, colon cancer, melanoma, osteosarcoma, glioblastoma, and lung cancer. As expected, differentiated cancer cells showed decrease in expression in cancer promoting proteins, malignant features and tumorigenicity. This was the first piece of evidence that, in contrast to extensive heterogeneity, different cancer cells might share the property of neural stem/progenitor cells, i.e., neural stemness. In agreement, expression of the genes for these epigenetic factors are all enriched in embryonic neural cells during early neural development, and they play essential role in maintaining stemness of neural stem cells (Zhang et al., 2017; Cao, 2017). Further analysis revealed that the “core EMT factors/markers,” SNAI1/2, ZEB2, TWIST1, N-Cadherin and Vimentin, which promote cancer or generally upregulated in cancer cells, are also specifically expressed or enriched in embryonic neural cells. In fact, these are markers for neural stem/crest cells. It seemed that embryonic neural expression of cancer promoting genes should not be just accidental. To clarify whether it is a general rule that cancer promoting genes are embryonic neural/neural stemness genes, more than 3,000 cancer genes were categorized into genes promoting cancer/upregulated in cancer cells, genes suppressing cancer/downregulated in cancer cells, and genes playing dual roles in cancer, and their embryonic tissue expression was analyzed. The analysis led to the generalization that most (if not all) cancer promoting genes are embryonic neural/neural stemness genes, and by contrast, a majority of cancer suppressor genes are non-neural genes (Zhang et al., 2017). Therefore, cancer cells share regulatory networks with embryonic neural cells, which confer cancer cells the property of neural stemness (Zhang et al., 2017; Cao, 2017). The link between neural stemness and cancer cells is also manifested by many lineage-tracing studies. For example, CD133 (or PROM1), Msi1, Sox2, and Dclk1 were used as tracing markers to identify cancer-initiating cells or cancer stem cells (CSCs) in different types of cancer, including colon cancer, pancreatic cancer, squamous-cell carcinoma (Boumahdi et al., 2014; Fox et al., 2016; Nakanishi et al., 2013; Ricci-Vitiani et al., 2007). CD133, Sox2, Msi1 or Dclk1, which are frequently used as CSC markers, are either typical markers for NSCs and/or their genes are specifically expressed in neural tissues in vertebrate embryos. Considering that neural stemness represents the ground state for cell differentiation, neural stemness and expression of genes involved in promoting/maintaining neural stemness genes is diluted in differentiated cells. Differentiated cells in postnatal animal/human may experience intracellular/extracellular insults, including mutations, chromosomal instability, aneuploidy, microenvironmental changes, gene misregulation, etc., which may accidentally cause downregulation/silencing of tissue-specific genes or differentiation genes, or upregulation/activation of neural genes or both. Then differentiated cells will return progressively back to their original ground state, i.e., neural stemness. These results shed the light on the unified principle underlying tumorigenesis beyond enormous inter- and intra-tumoral heterogeneities: tumorigenesis represents a process of progressive loss of original cell identity and acquirement of neural stemness in postnatal animal/human cells along the default route determined by evolution (Cao, 2022; Cao, 2017) (Figure 2). Such a paradigm has been validated by increasing studies. For instances, loss of a transcriptional repressor causes transition of intestinal stem cells into NSC-like state and drives neuroendocrine tumor formation (Li et al., 2020); dedifferentiation of neuron into a neural stem-like state initiates tumorigenesis (Southall et al., 2014); Tuft cells transdifferentiate to neural-like progenitor cells during progression of pancreatic cancer (Salas-Escabillas et al., 2025); loss of muscle differentiation factor Myod1 leads to gain of neural stemness and tumorigenicity in myoblasts (Xu et al., 2021); neural-like dedifferentiation in cancer cells was observed during melanoma tumorigenesis (McGrail et al., 2025). Single cell RNA sequencing data also revealed dedifferentiation from the melanocytic toward the neural crest-like state during uveal melanoma progression (Xu et al., 2025), malignant cells with a neural crest-like state during gliomagenesis (Hamed et al., 2025), and neural cell state in different cancer cells (Pascua et al., 2021; Xing et al., 2025). Because neural stemness is the general stemness, from which other stemness (including ESC stemness) and differentiated cells are derived, it is comprehensible that tissue stemness is closer to neural stemness than fully differentiated cells. The higher is the differentiated status of a cell, the farther away it is from neural stemness. Therefore, tissue stem cells are more susceptible to neoplasmic transformation than fully differentiated cells because they are closer to neural stemness.

Cancer cells share not only regulatory networks with neural stem/embryonic neural cells, but also various phenotypic traits, such as single-cell migration (Cao, 2022; Zhang et al., 2017; Cao, 2017). Both are tumorigenic, defined by or dependent on activation of ancestral regulatory networks, and prone to genomic instability. Both exhibit neural stemness and pluripotent differentiation potential. Their metabolism is characterized by aerobic glycolysis. Both cancer genes and genes defining neural stemness are characteristic of over-representation of long genes with more exon/intron compositions that facilitate generation of more splice variants. Cancer cells are characteristic of neural stemness, which has a unicellular origin (Cao, 2022; Xu et al., 2021; Zhang et al., 2017). This agrees with that cancer cells are formed via a process of reverse evolution, i.e., multicellular to unicellular state, and cancer cells are characteristic of unicellular-like state (Alfarouk et al., 2011; Anatskaya et al., 2020; Chen et al., 2015; Vinogradov and Anatskaya, 2025) (Figure 2). Both cancer cells and neural stem cells are immune privileged cells. Embryonic pluripotent cells also share most of these features because the default fate of pluripotent cells is neural stemness and pluripotency has a unicellular origin (Cao, 2022).

### 3.2 Neural stemness as the source of cell tumorigenicity

Among different types of stem cells, ESCs and iPSCs are tumorigenic, but tissue stem cells, such as hematopoietic stem cells, mesenchymal stem cells, are not. NSCs were considered as a type of tissue stem cells and hence non-tumorigenic, although there were sporadic reports about tumorigenic potential of primitive NSCs derived from ESCs or iPSCs. It was routinely explained by incomplete change of ESCs or iPSCs into NSCs or by the expression of MYC oncoprotein in iPSCs (Deng et al., 2018a; Germain et al., 2012). The standard *in vivo* tumorigenicity assay is xenograft tumor formation in immunodeficient mice. My studies demonstrated that either primitive NSCs derived from ESCs, NSCs from E9 mouse embryos, and neural progenitor cells isolated from cortices of E13.5 mouse embryos were able to form xenograft tumors in immunodeficient mice. By contrast, loss of neural stemness via differentiation of NSCs into neuronal cells leads to reduced tumorigenicity (Xu et al., 2021; Chen et al., 2021). Myoblast cells are not tumorigenic. Loss of Myod1 causes acquirement of neural stemness and tumorigenicity, and loss of neural stemness via differentiation into neuronal cells also causes reduced tumorigenicity (Xu et al., 2021). These are the direct evidence that neural stemness determines tumorigenicity of cells. ESCs in absence of TGFβ signaling, which suppresses tumorigenesis, adopt their default fate, i.e., the primitive NSCs. Correspondingly, ESCs are less tumorigenic than primitive NSCs (Xu et al., 2021). In combination with the evolutionary advantage of neural genes and neural stemness and the emergence of TGFβ signaling at the start point of multicellularity during evolution, it can be concluded that neural stemness, but not other cellular properties/states, is the cellular property conferring tumorigenicity in cells (Cao, 2022; Xu et al., 2021; Cao, 2017).

**FIGURE 3:**
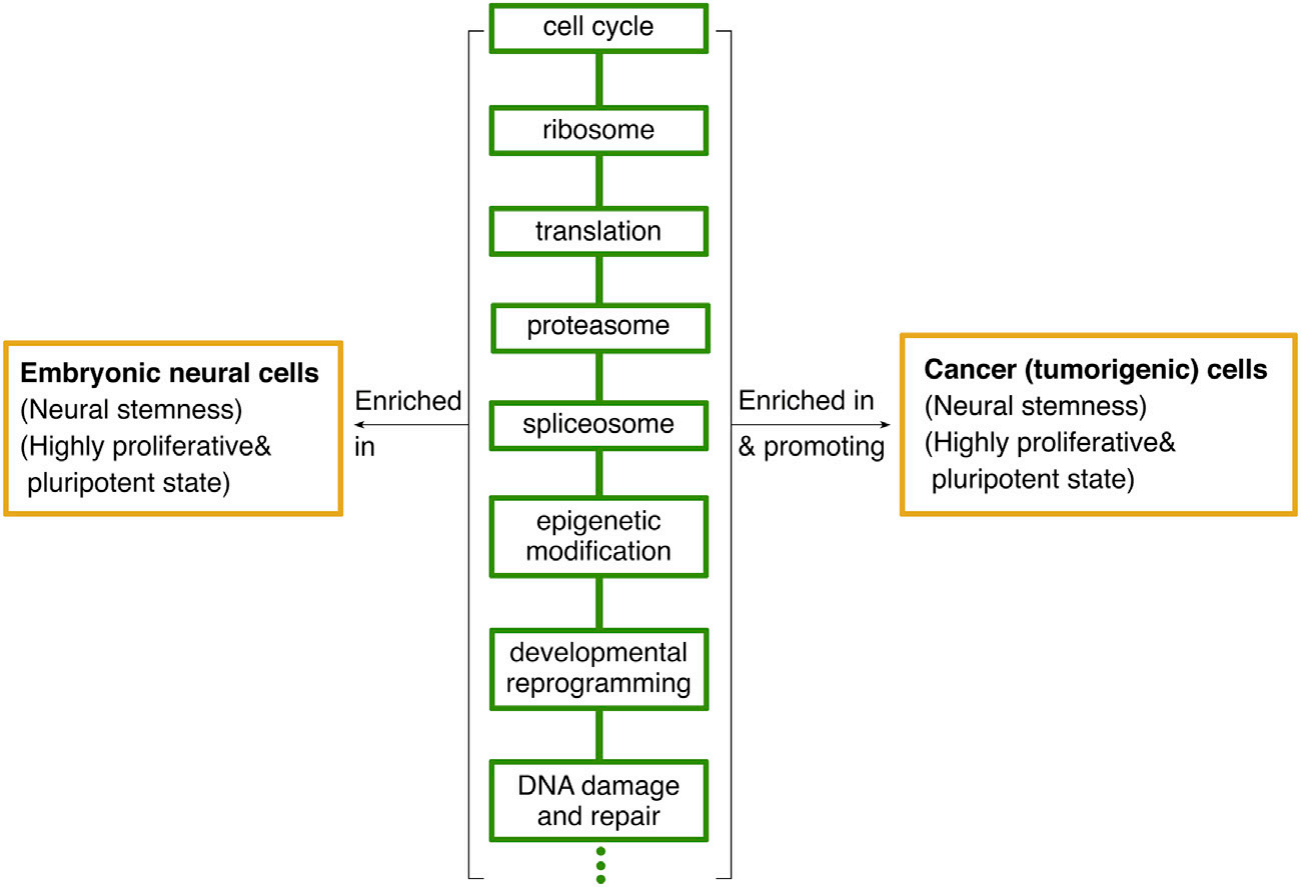
Basic cellular machineries are enriched in both embryonic neural cells and cancer cells. These basic machineries work concerted together to define a cellular state with fast proliferation and pluripotency, serving as a basal state from which other cell types can be derived.

As mentioned above, genes coding for the components of machineries required for basic cellular physiological functions and developmental programs, such as cell cycle, ribosome, spliceosome, proteasome, epigenetic modification, reprogramming, DNA damage and repair, are mostly enriched in embryonic neural cells. These machineries are also enriched in cancer cells and play roles in promoting cancer. Cancer cells are characteristic of fast cell cycle/proliferation. They need more protein syntheses to sustain cell growth, more protein turnover program to maintain protein homeostasis; they need more DNA synthesis, and hence more DNA damage and repair mechanism; they undergo differentiation, hence they need more proteins involved in developmental programs, and so on. These machineries work concerted together to define neural stemness in neural stem/embryonic neural cells, and define tumorigenic property in cancer cells (Cao, 2022; Chen et al., 2021) (Figure 3).

### 3.3 Pluripotency and tumorigenicity: two sides of a same coin

Pluripotency and tumorigenicity are usually considered as distinct cellular properties because they are the most fundamental cellular properties dictating embryogenesis and tumorigenesis, separately. However, numerous studies on pluripotency have implied the intrinsic link between the two cellular properties. The earliest evidence was the pluripotent differentiation potential of embryonal carcinoma cells derived from teratocarcinoma, a type of cancer that can originate from many types of tissues/organs. After identification of embryonal carcinoma cells in more than a decade later, mouse ESCs were isolated and the property of ESCs were observed to be very comparable with those of embryonal carcinoma cells. Both cell types display pluripotent differentiation potential because they form teratomas in immunodeficient mice and contribute to formation of chimeric embryos (Solter, 2006). Teratoma formation in immunodeficient mice is a standard assay of pluripotency. Xenograft tumor formation performed in the same way is a standard assay of tumorigenicity. Histologically identifiable tissues/organs from different germ layers that are visible in teratomas formed by pluripotent cells, such as nerves, gut and glandular tissues, and cartilaginous tissues, are usually not visible in xenograft tumors formed by cancer cells. However, cell types that are derived from all three germ layers are present in the tumors (Xu et al., 2021; Zhang et al., 2022). This means that xenograft tumors are degenerated forms of teratomas. Besides teratocarcinoma cells, a variety of other cancer cells, including leukemia, neuroblastoma and melanoma cells, can contribute to chimeric formation or be induced to differentiate into different types of cells when transplanted into an embryo. The differentiated offspring cells are similar to host cells and not tumorigenic anymore (Brinster, 1974; Cooper and Pinkus, 1977; Gerschenson et al., 1986; Gootwine et al., 1982; Hendrix et al., 2007; Illmensee and Mintz, 1976; Kulesa et al., 2006; Papaioannou et al., 1975; Podesta et al., 1984; Webb et al., 1984; Wells and Miotto, 1986). Moreover, transplantation of the nuclei of different cancer cells into enucleated oocytes led to development of normal embryos (DiBerardino et al., 1983; Hochedlinger et al., 2004; Li et al., 2003; King and DiBerardino, 1965; McKinnell et al., 1969), suggesting the pluripotent nature of cancer cells. Characterization of cancer cells and NSCs demonstrates that variants of pluripotent state can be numerous and are present throughout the life of an animal/human, from a pre-implantation blastocyst to adult stage. It can be seen that, historically (actually, not very long ago), cancer cell was the first cell that was characterized as the cell with pluripotency, which later on inspired the study of ESC pluripotency. Pluripotent cells can differentiate into various intermediate states, finally into fully differentiated cells. Therefore, pluripotency has become the cornerstone of developmental/stem cell biology, and inspired understanding of phenotypic complexity during embryogenesis. Ironically, the pluripotent property of cancer cell has faded into oblivion in cancer research, generating no illumination of understanding the phenotypic complexity/plasticity in cancer. Instead, phenotypic alteration of cancer cells during tumorigenesis has been primarily understood with “EMT” (Bakir et al., 2020; Chaffer et al., 2016; Kalluri and Weinberg, 2009; Lu and Kang, 2019; Nieto et al., 2016), a poorly defined concept in which no basic scientific rationale can be found (Cao, 2023; Cao, 2024; Yang et al., 2020; Cao, 2017).

**FIGURE 4:**
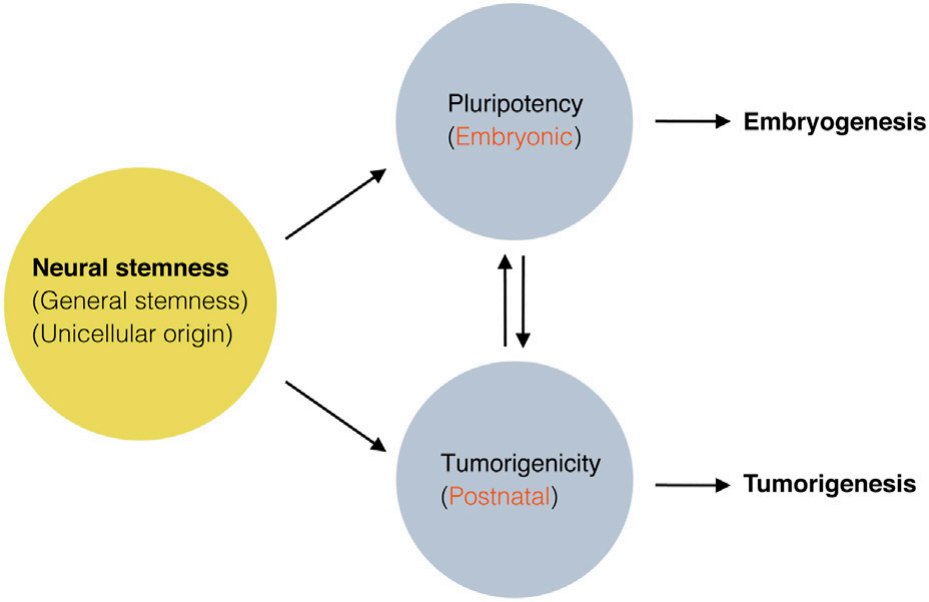
Relationship between the basic cellular properties. Evolutionary advantage of neural stemness, together with neural state as the default state of embryonic pluripotent cells and neural stemness as the core property of cancer cell, predetermines that neural stemness is the general stemness and dictates both pluripotency and tumorigenicity, the basic cellular properties controlling embryogenesis and tumorigenesis, respectively. Pluripotency and tumorigenicity are both but different manifestations of a same cellular property, i.e., neural stemness, during embryonic and postnatal stages of animal life, respectively. Without neural stemness, embryonic developmental process cannot continue and tumorigenesis cannot occur and progress.

Pluripotency and tumorigenicity are both determined by neural stemness, implying that they are coupled cellular properties. Experimental evidence showed that it is the case. Blocking an endogenous factor, which promotes neural stemness and cancer, in neural stem cells and cancer cells led to a neuronal differentiation effect and loss of neural stemness. The resulting cells showed a simultaneous decrease in both tumorigenicity and pluripotency. Vice versa, enhancing neural stemness in cancer cells caused a simultaneous increase (Zhang et al., 2022). Pluripotency manifested by chimeric formation means that, in the presence of embryonic inducing factors, pluripotent cells, including cancer cells, NSCs and embryonic pluripotent cells, can be induced to differentiate into normal cells in an embryonic milieu and integrated into the development of an embryo. In a postnatal animal/human, they differentiate and form tumor structures that cannot be integrated into normal differentiated tissues/organs because of no correct embryonic differentiation signals. The different behavior of pluripotent cells in embryonic milieu and in a postnatal animal/human suggests that tumorigenicity is actually the manifestation of pluripotent state in a postnatal animal/human. In summary, pluripotency and tumorigenicity are both but different manifestations of the same cellular property, neural stemness, in embryonic and postnatal stage of animal/human life, respectively (Figure 4).

### 3.4 Neural stemness or general stemness represents cancer stemness

CSC is an important concept in cancer biology because it is believed that CSCs are capable of differentiation, thereby contributing to TME. However, properties of CSCs and their regulatory network have been poorly characterized. Cancer stemness is characteristic of some common features or hallmarks, including self-renewal and differentiation, multipotency, tumor initiation, immune evasion, etc. (Agudo and Miao, 2025; Loh and Ma, 2024). Critical questions still remain. Answers to these questions are essential for understanding the property of cancer cells, TME formation, and for development of novel strategies for cancer therapy. It cannot be determined or inferred from these criteria whether CSCs from different types of cancer share a common type of stemness, or CSCs of different cancer types exhibit the tissue stemness of their respective tissues of cancer origin, or CSCs are not comparable with any known stem/progenitor cell types (Cao, 2022). CSCs are isolated by using a few specific surface markers (Agudo and Miao, 2025). This would lead to pinpointing only a subset of CSC populations because of high heterogeneity of CSCs, and thus leading to misinterpretation of the properties of cancer stemness. Identification of CSCs also relies on approaches commonly used for identifying adult tissue stem cells. Moreover, CSCs are thought to be comparable with tissue stem cells because CSCs and some, but not all, tissue stem cells are immune privileged (Agudo and Miao, 2025). This implies that CSCs are characteristic of adult tissue stemness, a premise that has not been confirmed. If this is true, it can be deduced that CSCs of different types of cancer should have the differentiation potential similar to their respective tissue stem cells. However, there is no such evidence to show lineage-specific differentiation hierarchy of CSCs of a particular cancer (Cao, 2023; Cao, 2022). By contrast, CSCs show multi-lineage differentiation capacity, such as CSCs in colon cancer (Cao, 2022; Vermeulen et al., 2008). Vice versa, there is also no evidence to show that any type of adult tissue stemness reveals the features of tumor initiation, metastasis, or therapy resistance, which are the hallmarks of CSCs (Loh and Ma, 2024), and no evidence to show that CSCs share the regulatory networks with certain adult tissue stem cells. EMT explains that cancer stemness is a consequence of acquirement of mesenchymal state in cancer cells (Celià-Terrassa and Jolly, 2020; Dongre and Weinberg, 2019; Tanabe, 2022). It is hard to understand how an unknown and indefinable “mesenchymal state” can help to understand cancer stemness (Cao, 2023; Cao, 2024; Yang et al., 2020; Cao, 2017). The analyses above revealed that cancer (tumorigenic) cells share regulatory networks, cellular properties and even evolutionary advantage with neural stem/embryonic neural cells, clarifying that neural stemness or general stemness, but not other types of stemness, represents cancer stemness. As cancer progresses, neural stemness of cancer cells will progressively increase, meaning the enhancement in differentiation potential or plasticity (Zhang et al., 2022; Hunter et al., 2025; Moorman et al., 2025).

### 3.5 Neural stemness unifies phenotypic traits of cancer cells

**FIGURE 5:**
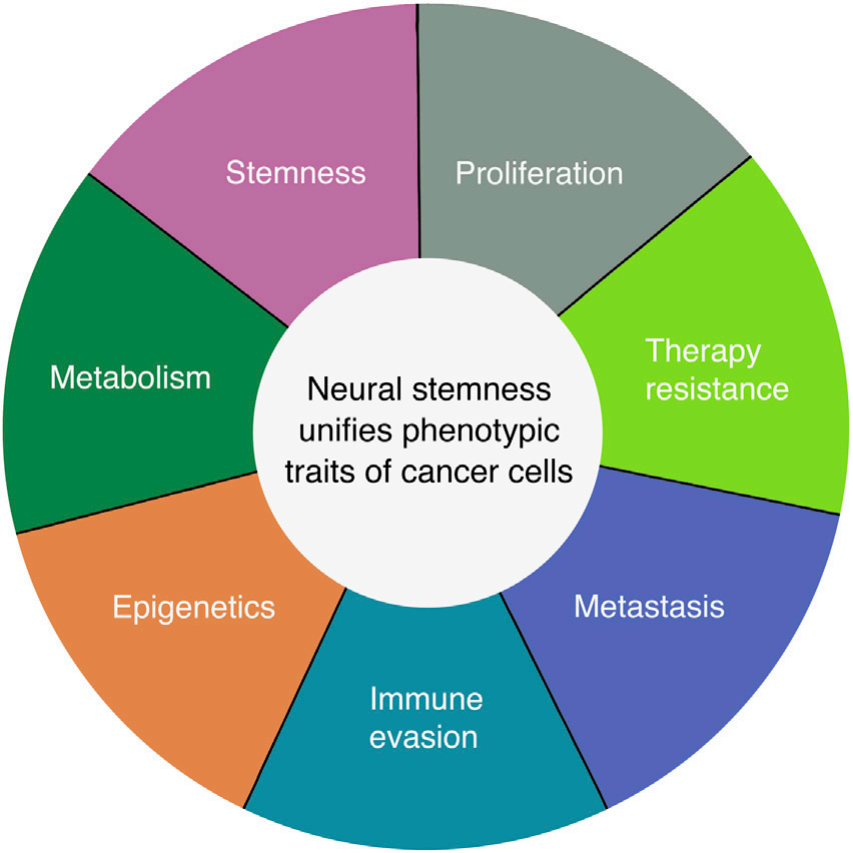
Unification of phenotypic traits of cancer cell by neural stemness. Regulatory networks of different phenotypic traits of cancer cell are part of regulatory networks of neural stemness.

Cancer cells display some phenotypic traits, such as stemness, high proliferation, invasion/migration, evasion of death and anti-cancer immunity, dysregulated metabolism and epigenetics, therapy resistance. Predominant research of cancer cell biology seems to be the regulation of a particular trait by a specific gene/factor, creating an impression that different traits of cancer cells are independent from each other. It should not be the case. Major cancer promoting factors are best investigated for their roles in regulating cancer cell phenotypic traits. It is common to see that one factor is able to regulate different traits of cancer cells. For an instance, the epigenetic factor EZH2 plays oncogenic role in different types of cancers, promotes cancer cell stemness (Balinth et al., 2022), proliferation (Bryant et al., 2007), metastasis (Zingg et al., 2015), chemoresistance (Crea et al., 2012; Ougolkov et al., 2008), metabolism (Ahmad et al., 2017), etc. Of course, its upregulation in cancer cells leads to global change in epigenetic modification in genome and proteins. Similarly, studies on the oncoprotein C-MYC revealed that it regulates almost all traits of cancer cells (Dhanasekaran et al., 2022; Fatma et al., 2022; Llombart and Mansour, 2022). Cancer immunity and metabolism are prevailing research fields in cancer biology. Typical oncoproteins that play multiple roles in cancer are also major regulators of cancer cell metabolism and immunogenicity, e.g., EZH2 (Kim et al., 2020; Zhou et al., 2020; Nylund et al., 2021), C-MYC (Miller et al., 2012; Zimmerli et al., 2022), KRAS (Kerk et al., 2021; Watterson and Coelho, 2023; Lasse-Opsahl et al., 2025). A same factor being able to regulate different phenotypic traits of cancer cells suggests that these traits are intrinsically interconnected. SNAI1/2, ZEB2 and TWIST1 could serve as an additional example. These “core EMT factors” have not been and cannot be clarified to be specific markers for “mesenchymal state,” because the cellular state is unknown and indefinable (Cao, 2024; Yang et al., 2020). Instead, they are markers for neural stem or neural crest cells, and their roles in regulating neural stemness are well documented. They were initially employed to explain single-cell migration of cancer cells in the context of the “EMT concept.” In addition to their role in promoting cancer cell migration, they also regulate or are regulated by many other cancer promoting factors that are involved in regulation of almost all phenotypic traits of cancer cells, including stemness, proliferation, therapy resistance, metabolism, epigenetics, immune evasion (Cao, 2024). All these cancer promoting factors are components of embryonic neural regulatory network, which endows cancer cells with neural stemness (Cao, 2022; Xu et al., 2021; Zhang et al., 2022; Zhang et al., 2017; Cao, 2017). This means that different phenotypic traits of cancer cells are ultimately determined and coupled together by neural stemness and its corresponding regulatory networks, similar to the unification of tumorigenicity and pluripotency of cancer cells by neural stemness (Zhang et al., 2022) (Figure 5). Disrupting one phenotypic trait will inevitably affect one or more, if not all, other traits of cancer cells.

### 3.6 Neural stemness and immune privilege of cancer cells

Cancer cells are tumorigenic and immune privileged cells that are capable of immune evasion. How tumorigenicity and immune evasion are correlated was major topic to investigate. A relevant research focus on cancer immunotherapy is to find out ways to boost the sensitivity of cancer cells to anti-cancer immunity. Extensive studies on major oncoproteins have revealed their critical roles in promoting immune evasion and immunotherapy resistance of cancer cells via pairwise molecular regulatory mechanisms. Here are some examples. C-MYC suppresses STING-IFN signaling, thereby weakening immune cell infiltration in triple-negative breast cancer (Zimmerli et al., 2022); Inhibition of KRAS(G12D) induces FAS expression in cancer cells and facilitates CD8^+^ T cell-mediated death (Mahadevan et al., 2023); Inhibition of EZH2 upregulates MHC class I expression, leading to increase of antigen-specific CD8^+^ T-cell proliferation, IFNγ production, and tumor cell cytotoxicity (Zhou et al., 2020); Inhibition of CDK4/6 enhances T cell activation as a result of de-repression of NFAT family proteins (Deng et al., 2018b); beta-catenin represses *CCL4* transcription, thus inhibiting anti-cancer immunity (Spranger et al., 2015); HDAC1 brakes anti-cancer immunity via suppression of type I dendritic cell maturation (De Sá Fernandes et al., 2024). These pairwise regulatory mechanisms might be countless because both cancer and immunity are complex. Still, “oncogenic signaling is the least understood aspect of functional immunogenicity” (Karasarides et al., 2022). The molecular mechanisms above appear nothing in common. When considering the fact that most cancer promoting proteins are embryonic neural proteins, including C-MYC, EZH2, KRAS, beta-catenin, CDK4/6, and HDAC1, it can be deduced that it is a cellular property, i.e., neural stemness, that is the ultimate factor to determine immune evasion. Cancer stemness is considered as a key factor driving immune evasion and immunotherapy resistance (Agudo and Miao, 2025; Galassi et al., 2021). The link now becomes clear because neural stemness represents cancer stemness.

My latest research revealed that the link between neural stemness and cancer cell immune evasion should be understood according to principles of developmental biology (Liu et al., 2025). Neural stemness is determined by genes specifically expressed in or enriched in neural stem cells/embryonic neural cells, which are generally repressed or silenced in non-neural cells. Vice versa, genes specifying non-neural cells or maintaining their identities/functions, including immune related genes, are not highly expressed in embryonic neural cells. Interestingly, genes enhancing immunogenicity, including IFN-γ response genes and genes involved in antigen processing and presentation, are not or only weakly expressed in neural stem or embryonic stem cells, but they are highly expressed in immune cells and other non-neural cells, e.g., muscle and fat cells. The difference in expression of immune related genes is in agreement with that neural stem cells and embryonic stem cells, whose default fate is neural stem cells, are immune privileged, but other types of cells are not (Fändrich et al., 2002; Hori et al., 2003; Drukker et al., 2006; Magliocca et al., 2006; Itakura et al., 2017; Ozaki et al., 2017). This means that neural stemness endows cancer cells with tumorigenicity, pluripotency and other malignant features, and immune privilege as well. In general, most (if not all) oncoproteins are embryonic neural proteins and play roles in specifying neural stem/precursor cells and/or maintaining neural stemness. On one hand, they promote immune evasion by promoting the cancer regulatory network, i.e., the embryonic neural network, and on the other they are involved in repressing non-neural genes, including immune related genes in neural stem cells and cancer cells. Induced differentiation of cancer cells, either by forced expression of a lineage-specific differentiation factors or by inhibition of endogenous cancer promoting factors, led to reprogramming the regulatory networks of cancer cells into those of differentiated cells (Liu et al., 2025; Ascic et al., 2024; Linde et al., 2023; Zimmermannova et al., 2023), and reprogramming the cellular properties of cancer cells into the properties of differentiated cells with reduced neural stemness and tumorigenicity. Differentiation also generates the general tendency of decreased expression of cancer promoting genes, which promotes immune evasion, and increased expression of immune related genes including those enhancing immunogenicity, and enhancing immunogenicity of cancer cells (Liu et al., 2025; Ascic et al., 2024; Linde et al., 2023; Zimmermannova et al., 2023). In summary, neural stemness confers cancer cells with the capability of immune evasion. Tumorigenicity and immunogenicity are inversely correlated properties of cancer cells.

**FIGURE 6:**
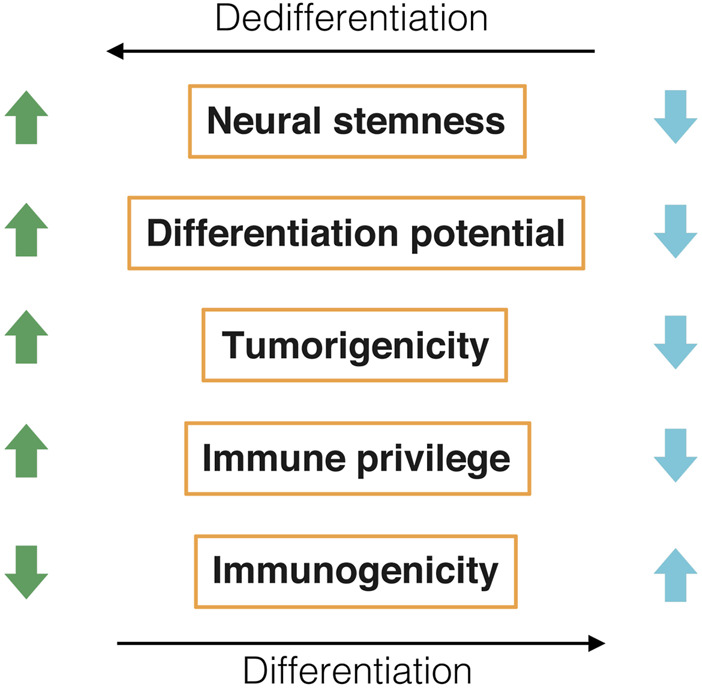
Coordinated but not independent change in different features of cancer cell. Tumorigenesis means a progressive process of dedifferentiation from a differentiated state to a neural ground state or neural stemness following the default route that is determined by evolution (See Figures 1, 2). This process is accompanied by increase in differentiation potential or plasticity, tumorigenicity, immune privilege, and hence decrease in immunogenicity in cells because of activation/upregulation of embryonic neural/neural stemness genes and silencing/downregulation of differentiation, tissue-specific and immune related genes. Vice versa, if cancer cell is induced to differentiate, an opposite effect of coordinated change in cell features will occur.

Analysis above revealed the shared characteristics between neural stem/progenitor cells and cancer cell, indicating that neural stemness determines phenotypic traits of cancer cells (Table 1). Tumorigenesis represents a progressive process of dedifferentiation, during which cells lose their original cell identity and acquire neural stemness, and accordingly, acquire tumorigenicity, pluripotent differentiation potential, immune privilege and lose immunogenicity. Upon differentiation, cancer cells lose their neural stemness, and hence tumorigenicity, pluripotency, immune privilege and acquire immunogenicity (Figure 6). Such a relationship suggests a strategy of cancer therapy (See text below).

**TABLE 1:** Comparison of cellular properties between neural stem/progenitor cells, cancer cells and pluripotent stem cells.

Neural stem/progenitor cells (references)	Cancer cells (references)	Pluripotent stem cell (PSCs) (references)
Tumorigenic (Xu et al., 2021)	Tumorigenic	Tumorigenic (Ben-David and Benvenisty, 2011)
Migratory	Migratory	Migratory
Immune privileged (Hori et al., 2003; Itakura et al., 2017; Ozaki et al., 2017)	Immune privileged	Immune privileged (Fändrich et al., 2002; Drukker et al., 2006; Magliocca et al., 2006)
Defined by ancestral regulatory networks (Xu et al., 2021; Domazet-Loso et al., 2007)	Dependent on activation of ancestral regulatory networks (Bussey et al., 2017; Domazet-Loso and Tautz, 2010; Trigos et al., 2017; Trigos et al., 2018)	Unknown
Neural stemness	Neural stemness (Cao, 2022; Xu et al., 2021; Zhang et al., 2022; Chen et al., 2021; Zhang et al., 2017; Cao, 2017; Lei et al., 2019)	Neural stemness as the default state of PSC (Tropepe et al., 2001; Muñoz-Sanjuán and Brivanlou, 2002; Smukler et al., 2006; Malaguti et al., 2013; Ying et al., 2003)
Pluripotent differentiation potential (Clarke et al., 2000; Tropepe et al., 2001; Xu et al., 2021)	Pluripotent differentiation potential (Xu et al., 2021; Zhang et al., 2022; Papaioannou et al., 1975; Mintz and Illmensee, 1975)	Pluripotent differentiation potential
Characteristic of aerobic glycolysis. Differentiation into neurons decreases glycolysis (Kim et al., 2014; Zheng et al., 2016)	Characteristic of aerobic glycolysis	Characteristic of aerobic glycolysis. Turning into NSCs does not change or increases glycolysis; differentiation into mesoderm and endoderm decreases glycolysis (Zheng et al., 2016; Intlekofer and Finley, 2019)
Unicellular origin (Cao, 2022; Xu et al., 2021)	Resulting from loss of original cell identity and acquirement of neural stemness, and reverse evolution from multicellular to unicellular state (Cao, 2022; Xu et al., 2021; Alfarouk et al., 2011; Anatskaya et al., 2020; Chen et al., 2015; Vinogradov and Anatskaya, 2025; Bussey et al., 2017)	Unicellular origin of pluripotency (Sogabe et al., 2019)
Prone to genomic instability (Varela et al., 2012)	Genomic instability	Prone to genomic instability (Peterson and Loring, 2014)
Enriched in long genes with more splice variants (Xu et al., 2021; Gabel et al., 2015; Zylka et al., 2015)	Enriched in long genes with more splice variants (Sahakyan and Balasubramanian, 2016)	Unknown
Enriched in basic machineries, e.g., those of cell cycle, ribosome biogenesis, spliceosome assembly, proteasome, epigenetic modification, developmental reprogramming, DNA damage and repair, etc. They work concertedly together to define a basal cellular state with high proliferation and pluripotency (Cao, 2022; Xu et al., 2021; Chen et al., 2021)	Enriched in basic machineries, e.g., those of cell cycle, ribosome biogenesis, spliceosome assembly, proteasome, epigenetic modification, developmental reprogramming, DNA damage and repair, etc. They play diverse roles in promoting cancer (Cao, 2022; Xu et al., 2021; Chen et al., 2021)	Unknown

## 4 Neural stemness unifies embryogenesis and tumorigenesis

Cancer was proposed as a disorder of developmental dynamics (Rubin, 1985). How the complex process of embryogenesis is intrinsically linked with the complex process of tumorigenesis has remained a challenging question, particularly when considering that normal embryogenesis needs fusion of gametes. However, the central role of neural stemness contributing to both pluripotency and tumorigenicity (Figure 4), the key cellular properties in developmental biology and cancer biology, respectively, immediately reminds of the intrinsic association between embryonic development and tumorigenesis. Before understanding the association, it needs to look back again on a paramount research work in developmental biology in history.

### 4.1 Embryonic neural induction, body axis formation and embryogenesis

**FIGURE 7:**
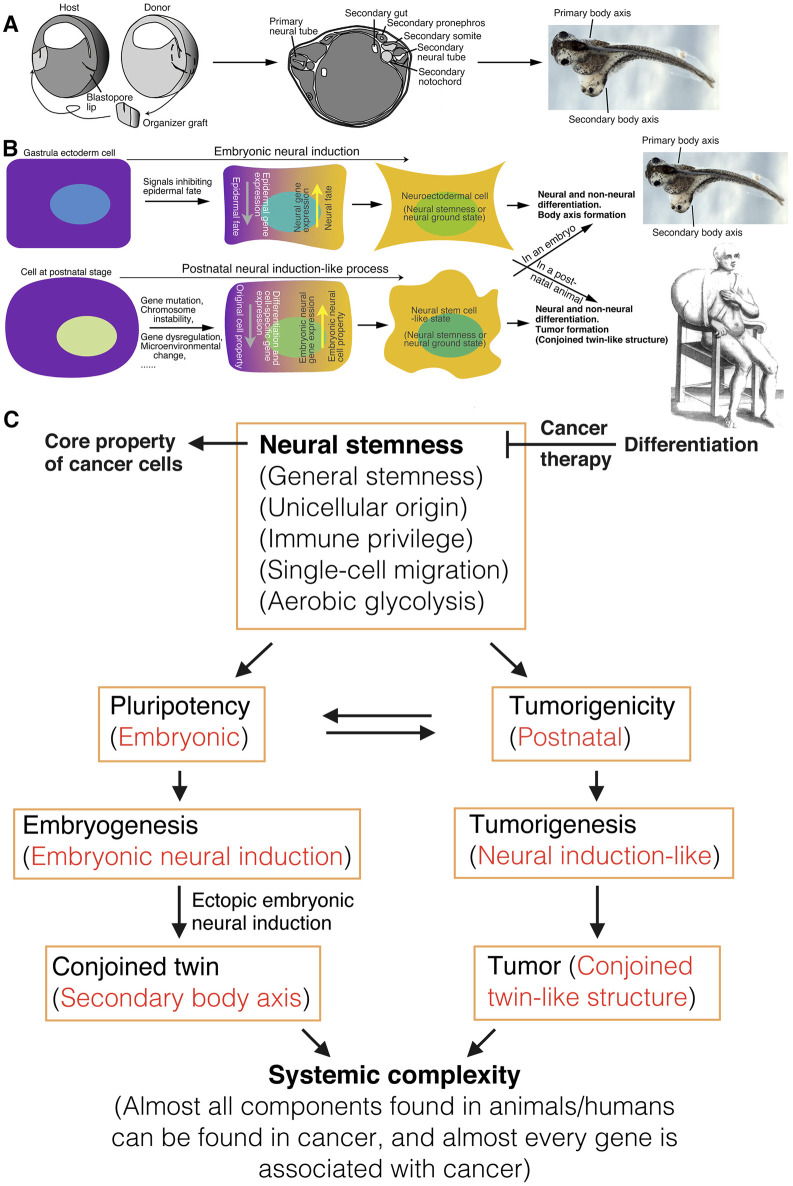
Neural stemness unifies embryogenesis and tumorigenesis. **(A)** (Left) A schematic illustration depicting the organizer grafting experiment done by Spemann and Mangold, in which the organizer (the dorsal blastopore lip) of an early gastrula of a light-gray newt (*Triturus cristatus*) was grafted to the site opposite to the dorsal lip of an early gastrula of a dark-gray newt (*Triturus taeniatus*). (Middle) An illustration showing secondary axis (or conjoined twin) formation by organizer grafting. A section through the trunk of a twinned embryo demonstrated that the graft contributed to the notochord, medial somites and floor plate of the secondary body axis, but the secondary neural tube, somites, pronephros, and archenteron cavity were induced from the host. (Right) Conjoined twin formation by Spemann-Mangold organizer grafting performed at early gastrula stage of frog (*Xenopus laevis*) embryo. **(B)** Acquirement of neural stemness is prerequisite for both embryogenesis and tumorigenesis. (Upper) Acquirement of neural stemness via embryonic neural induction and body axis formation. During embryonic development, the organizer or node secretes proteins inhibiting epidermal fate of gastrula ectoderm, leading to acquirement of neural fate in ectoderm and formation of neuroectoderm (neural stemness). The neural induction process is required for differentiation of both the nervous system and many non-neural tissues that are necessary for body axis formation. Ectopic neural induction or neural induction-like event in an embryo causes formation of secondary embryonic structures or a conjoined twin. (Lower) Acquirement of neural stemness via a neural induction-like process in postnatal animal/human and tumorigenesis. Cells of a postnatal animal/human may suffer various extracellular (e.g., microenvironmental change) and/or intracellular (e.g., gene mutations) insults, which could, occasionally or due to “bad luck,” cause upregulation/activation of embryonic neural genes or/and downregulation/silencing of tissue-specific or differentiation genes. This results in progressive loss of original cell identity and acquirement of neural stemness along the default route pre-determined by evolution. The resulting cells are capable of self-renewal and differentiation into tissue/cell types of all three germ layers, and consequently, leading to formation of a conjoined twin-like structure, i.e., a tumor in a postnatal animal/human. The rightmost is the first clinical illustration of tumor, a large scapulohumeral tumor, in a book by a surgeon Marco Aurelio Severino published in 1632 (Hajdu, 2011). Embryonic pluripotent cells (ESCs, NSCs) and cancer cell are exchangeable. Both can contribute to chimera formation when put into an embryo but form xenograft tumors in a postnatal animal because of lack of embryonic inducing signals. **(A)** is adapted from Harland (2008), **(B)** is from Cao (2023); Hajdu (2011); and Harland (2008) with permission from publishers. **(C)** Diagram illustrating how to understand cancer as a systemic disease by understanding neural stemness as the core property of cancer cell and basic rules dictated by neural stemness. During embryogenesis, acquirement of neural stemness in early embryonic cells, i.e., embryonic neural induction, is required for neural development and body axis formation. Ectopic embryonic neural induction leads to formation of a secondary body axis, i.e., a conjoined twin. In postnatal animals, acquirement of neural stemness in cells leads to acquirement of pluripotency. The resulting cells, i.e., cancer cells, can self-renew and differentiate into various types of cells in an environment that lacks embryonic differentiation signals, thereby forming a tumor. Neural stemness is the cellular property that governs both embryogenesis and tumorigenesis, and tumorigenesis is the recurrence of embryonic neural induction in postnatal stage. Neural stemness as the core property of cancer cell and the major contributor to cancer complexity suggests that inhibition of neural stemness via differentiation should be a strategy of cancer therapy.

A major question in developmental biology was to understand how the nervous system is induced to form and body axis is established during embryogenesis. The most inspiring work was done by Spemann and Mangold (1924). They found that transplantation of the dorsal blastopore lip, which was named Spemann-Mangold organizer later, of a newt gastrula embryo to the ventral side of a host gastrula embryo was able to induce a complete secondary body axis or a conjoined twin. The secondary body axis contained neural tube, somites, pronephros and gut that were derived from the host, and the transplanted dorsal blastopore lip differentiated mostly into notochord (Spemann and Mangold et al., 1924; Spemann and Mangold, 2001). By contrast, an embryo without organizer activity forms only a “belly piece” that contains no neural and dorsal structures (Spemann, 1938; Gerhart, 2001; De Robertis, 2009; Sosa et al., 2019). These experiments demonstrated the critical role of the organizer activity in inducing neural tissue and body axis during embryogenesis (Figure 7A). The mechanisms underlying the induction of neural tissue and body axis by organizer began to be understood progressively until 6 decades after the dorsal blastopore transplantation experiment (De Robertis, 2009). It was concluded that neural fate is actually the default fate of blastula ectodermal cells, but epidermal fate is induced. The organizer promotes neural fate in ectoderm and dorsalization of primary germ layers by secreting a number of factors, such as Noggin, Chordin, and Cerberus, which inactivate the signaling pathways promoting epidermalization of ectoderm and ventralization of body axis, i.e., TGFβ and Wnt signaling (De Robertis, 2006; De Robertis and Kuroda, 2004; Harland, 2000; Anderson and Stern, 2016; Bouwmeester et al., 1996; Sasai et al., 1994; Smith and Harland, 1992). Disruption of TGFβ and Wnt signaling led to secondary axis formation, neuralization of ectoderm in absence of inducing factors, and rescue of ventralized embryos (Hemmati-Brivanlou and Melton, 1994; Glinka et al., 1997). Due to the epidermal inhibitory activity of the organizer, neural induction during embryogenesis is a process of loss of epidermal fate and acquirement of the fate neuroectodermal cells (Figure 7B), i.e., the primitive NSCs, in ectoderm along a default route determined by evolution. The pluripotent neuroectodermal cells further contribute to not only formation of the nervous system, but also differentiation of non-neural cells during establishment of body axis.

Functional homologue known as the node has been identified in embryos of all classes of vertebrates, such as fish, birds and mammals. Similar to the organizer, the node displays the inducing activity for neural development and body axis formation through conserved molecular mechanisms (Gerhart, 2001; Martinez Arias and Steventon, 2018). Neural induction means activation or upregulation of a spectrum of neural genes, forming regulatory networks that define embryonic neural tissues. Thus, the effect of neural induction and formation of secondary axis or conjoined twin can also be mimicked by ectopic activation of genes that are enriched in embryonic neural cells. For example, ectopic activation of *eed*, *yy1*, *ski*, *egfr*, *erbb2*, *erbb4*, or *gsn* in *Xenopus* or zebrafish embryos causes formation of a partial secondary body axis that contains neural and non-neural tissues (Amaravadi et al., 1997; Kanungo et al., 2003; Nie and Chang, 2006; Satijn et al., 2001). On the contrary, disruption of embryonic neural genes causes defects in neural and axial differentiation in mouse embryos, ultimately leading to developmental arrest at early stages (Berk et al., 1997; Britsch et al., 1998; Donohoe et al., 1999; Faust et al., 1995; Gassmann et al., 1995; Threadgill et al., 1995). As a general rule, disruption of embryonic neural genes almost always causes severe disruption of embryonic development.

Neural induction, a process leading to the effect of acquirement of neural stemness in ectoderm, is the paradigm for understanding how neural tissue and body axis are initiated to form during early embryogenesis. Neural induction or a similar effect might aberrantly occur and be associated with some most complex pathological phenomena. Similar to the result of blastopore lip transplantation experiment, if a secondary organizer-like activity occurs erroneously in a gastrulating embryo, such as a human embryo, a conjoined twin will form (Levin, 1999). Moreover, a neural induction-like process could also occur in cells of a postnatal animal or human, which would essentially lead to formation of a conjoined twin-like structure, i.e., a tumor.

### 4.2 Tumorigenesis as a neural induction-like process, and tumors as conjoined twin-like structures in postnatal animals/humans

The proposal that tumorigenesis represents the process of progressive loss of original cell identity and acquirement of neural stemness in postnatal cells reminds of neural induction during embryogenesis, i.e., the loss of epidermal fate and gain of neural stemness in ectoderm, which can cause formation of a conjoined twin if it occurs ectopically. Acquirement of neural stemness in cells means acquirement of pluripotency and tumorigenicity. In the microenvironment in a postnatal animal/human, cancer cells undergo proliferation and differentiation. However, differentiated cells cannot integrate into normal tissues/organs because of lack of embryonic differentiation signals. Consequently, a conjoined twin-like structure, i.e., a tumor that contains various types of cells, is formed (Figure 7B). A tumor being analogous to a conjoined twin-like structure can be best exemplified by teratocarcinomas, which can occur in many different types of tissues/organs. Teratocarcinomas are composed of tissues derived from all three germ layers, including undifferentiated neural epithelial tissue, differentiated nerves, gut and glandular tissues, cartilaginous and muscle tissues, similar to teratomas formed by pluripotent cells in immunodeficient mice. Unlike teratocarcinomas, other tumors that are diagnosed in most tissues/organs, e.g., lung, breast, colon cancer, usually do not contain well differentiated histologically identifiable tissues. But still, different types of cells that are derived from all three germ layers can be detected across tumors, for example, SOX1 or SOX2-expressing cells representing cells with neural stemness and derived from ectoderm, ACTA2-expressing cells derived from mesoderm, and AFP-expressing cells derived from endoderm (Cao, 2022; Xu et al., 2021; Zhang et al., 2022). These cells can be detected across a wide range of cancer types. A similar trend can be found in public databases, which show that a majority of transcripts and their protein products have low cancer specificity (www.proteinatlas.org) (Uhlen et al., 2017), for example, BMI1, CDH2, DCLK1, FGFR4, MSI2, and SMARCA4 that indicate neural stemness; MAP2, NEUROG2, and TUBB3 that indicate neuronal differentiation; AFP, FOXA3, GATA6, and KRT8 that indicate endodermal tissue differentiation; and ACTA1, ACTA2, COL1A1, FXR1, and MEF2D that indicate mesodermal tissue differentiation (Cao, 2023). Olfactory neuroblastoma and small-cell lung cancer are originated from distinct organs. Nevertheless, they are similar in molecular heterogeneity and lineage trajectories (Finlay et al., 2024). Recent research on pan-cancer single-cell transcriptomic atlas demonstrated that, as tumor progresses, tissues gradually lose tissue-specific healthy organizations and acquire a convergent cancerous ecosystem (Shi et al., 2025), strengthening the commonality of components and component interactions across different tumors (Figure 2). The common theme of cell differentiation in different tumors reflects that they follow rules similar to embryonic development, and tumors (including teratocarcinoma) are degenerated forms of conjoined twin-like structures. In fact, tumorigenesis resembles an ectopic neural induction process, leading to gain of neural stemness and hence pluripotency in cells, which then differentiate long different lineages via interactions between tumor cells and interaction between tumor and host cells (Figure 7B).

Tumors being comparable with a conjoined twin-like structure can be further supported by partial secondary axis (conjoined twin) formation resulting from ectopic activation of oncogenes in embryos, such as *eed*, *yy1*, *ski*, *egfr*, *erbb2*, *erbb4*, or *gsn* (Amaravadi et al., 1997; Kanungo et al., 2003; Nie and Chang, 2006; Satijn et al., 2001). Secondary axis formation was also observed when Hif-1α was activated in early embryos (MacColl et al., 2023). Ectopic activation of β-catenin in embryos generates twinned embryo is a classical finding in developmental biology (Funayama et al., 1995; Kelly et al., 1995). Mutations leading to stabilization and nuclear accumulation of β-catenin in cells are usually the cause of some cancers, e.g., colorectal cancer. Correspondingly, these mutations are able to induce formation of a twinned mouse embryo (Brickman and Burdon, 2002). These results confirmed that genes or mutations leading to tumorigenesis can also induce formation of a conjoined twin in embryos, reinforcing the intrinsic link between neural induction, conjoined twin formation and tumorigenesis. To add more support, transplantation of cancer cells into the appropriate position of a blastula stage of zebrafish embryo can induce secondary body axis formation (Hendrix et al., 2007). Secondary body axis or conjoined twin formation involves most genes and developmental programs for cell differentiation and tissue/organ formation, and involves complex interactions between tissues/organs within the conjoined twin, and interactions with the primary body axis. A conjoined twin at postnatal stages should also contain microbes and experience physiological/pathological effects, such as aging, senescence, and inflammation. Long history of cancer research has revealed that most genes are associated with cancer (de Magalhães, 2022), and nearly all elements found in an animal/human, including aging, senescence, inflammation, and even microbes, can be found in cancer. Meanwhile, complex interactions or crosstalks within the tumor, and interactions between tumor and the host, have been extensively documented. This magnitude of complexity of cancer is only comparable to the complexity at the level of an organism.

The analysis above revealed the intrinsic association between embryonic neural induction and tumorigenesis. The former means the loss of epidermal fate and acquirement of neural stemness, hence the pluripotency in ectodermal cells, leading to neural development and body axis formation during embryogenesis. The latter means the loss of original cell identity and acquirement of neural stemness, hence the tumorigenicity in postnatal cells, leading to the formation of a tumor. Pluripotency and tumorigenicity are exchangeable cellular properties, contributing to systemic complexity of embryogenesis and tumorigenesis, respectively (Figure 7C). Alignment of tumorigenesis with embryonic neural induction, hence conjoined twin formation, have been experimentally validated. This paradigm, in which neural stemness functions as the cornerstone and the causal factor, can hopefully interpret the complexity of tumorigenesis and cancer ecosystem as a whole. It is interesting to note that the paramount work on embryonic induction by Spemann and Mangold was almost never mentioned in cancer research. Instead, the epigenetic landscape by Conrad Waddington, who was a great admirer of Spemann and Mangold’s discovery of the principle of embryonic induction, has been frequently cited to interpret the link between tumorigenesis and embryonic development (Aranda-Anzaldo and Dent, 2018; Nicoglou, 2018).

## 5 Inverse correlation between cancer and neurodegeneration

Epidemiological evidence from large-scale longitudinal and cohort studies has consistently demonstrated an inverse correlation between cancer and neurodegenerative diseases, particularly Alzheimer’s disease (AD) and Parkinson’s disease (PD), meaning individuals with one condition are less likely to develop the other (Driver, 2014; Li et al., 2014; Zabłocka et al., 2021). Cancer and neurodegeneration represent opposite ends of a cellular spectrum: cancer involves uncontrolled proliferation and survival, while neurodegeneration features premature cell death and loss. Some research has elucidated that mechanisms including dysregulated pathways are operating in reverse directions in cancer and neurodegenerative diseases. For instance, the tumor suppressor p53 is downregulated in cancer to promote proliferation but upregulated in AD and PD, inducing neuronal apoptosis and tau hyperphosphorylation (Zabłocka et al., 2021). Pin1, a prolyl isomerase, is overexpressed in cancers to drive proliferation but inhibited in AD, resulting in pathogenic protein conformations like tau tangles and amyloid-beta accumulation (Driver, 2014). A latest study showed that peripheral cancer inhibits amyloid pathology and rescues cognition via secretion of an embryonic neural factor, cystatin-c (Cyst-C) (Li et al., 2026), Regulators of cell cycle, apoptosis or metabolism are also implicated in the inverse correlation. Nevertheless, more fundamental reason for the inverse correlation should be interpreted by the fact that the core property of cancer cells is neural stemness and cancer promoting genes are embryonic neural or neural stemness genes. This means cancer promoting genes play roles in all aspects of neural development and regeneration, including differentiation, migration, maturation, neuritogenesis, axonal guidance, etc. Cancer cells are capable of neuronal differentiation, secrete neurotrophic factors that are required for survival and functioning of nerves, including NGF, BDNF, Neurotrophin-3/4/5, GDNF, and release neurotransmitters (Li and Cho, 2011; Park and Lee, 2024).

Cancer therapies, particularly chemotherapy, radiation, hormone therapy, and emerging immunotherapies can generate a side effect of neurodegeneration in both children and adults by disrupting normal neural stem and precursor cell function, leading to ultimately neurocognitive deficits [often called “chemobrain” or “chemotherapy-induced cognitive impairment (CICI)”] in patients with tumors, including breast cancer, colorectal cancer, lymphoma, and brain tumors (Gibson and Monje, 2012; Joly et al., 2015; Lange et al., 2019; Yang and Moon, 2015). Although mechanisms involved in neuroinflammation, blood-brain barrier, etc., are proposed for CICI (Rao et al., 2022), the similarity between cancer and neural cells in both regulatory networks and cellular property is the most obvious connection between cancer therapies and therapy-induced neurodegeneration.

Numerous studies, as those mentioned above, have shown that cancer cells share regulatory networks and characteristics of neural (stem/progenitor) cells, are capable of multi-lineage including neuronal differentiation, secrete neurotrophic factors and release neurotransmitters, make connections with host nervous systems, and cancer is inversely correlated with neurodegeneration. Cancer-nervous system crosstalk has been extensively investigated and blocking the influence of host nervous systems on cancer progression is proposed to be the strategy of cancer therapy (Benzaquen et al., 2024; Mancusi and Monje, 2023; Silverman et al., 2021). However, neural property of cancer cells has drawn little attention. For cancer therapy, whether to block the host nervous systems or block the neural property of cancer cells is worth considering.

## 6 Important issues to consider or re-consider in cancer research

It’s no doubt that great progresses have been achieved in cancer research, which deepens essentially the understanding of cancer and leads to development of innovative cancer therapies, such as immune checkpoint inhibition. But “frustratingly, few of these innovative new therapeutic strategies are broadly beneficial across the spectrum of human cancers and, with many avenues for tumors to evolve resistance, even fewer significantly prolong overall survival” (Swanton et al., 2024). Development of efficient therapeutic strategies depends on the understanding of cancer cell. Nevertheless, scrutiny of some important “concepts” that are used as a basic tool to measure phenotypic traits of cancer cell, such as “EMT,” revealed that they are just misconceptions (Cao, 2023; Cao, 2024; Yang et al., 2020; Cao, 2017). In the context of “EMT,” loss of epithelial state, e.g., by loss of the epithelial protein E-cadherin, will inevitably cause transition to mesenchymal state, thereby promoting metastasis of cancer cells. A latest study (and also other previous research) showed no “EMT” but upregulation of embryonic neural factors GRPR and YAP1 after E-cadherin loss (Raymond et al., 2025). As a matter of fact, “EMT factors/markers” that are upregulated/activated during tumorigenesis, including SNAI1/2, ZEB2, TWIST1, N-cadherin, Vimentin, are also embryonic neural factors, which are mistakenly labeled as mesenchymal proteins/markers (Cao, 2024). The consequence is the misattribution of the key role of neural stemness in determining phenotypic traits of cancer cells to the indefinable “mesenchymal state,” “EMT” and its related cellular state transitions as groundless and scientifically meaningless concepts was ever discussed in details (Cao, 2023; Cao, 2024; Cao, 2017). Cancer cell is resulted from the change of cellular property and the change in expression of genes/proteins, and tumorigenesis is a highly flexible and dynamic process. Making correct association between gene/protein symbols and cellular state/property or making correct interpretation of expression of genes/proteins in cancer is critical for correct understanding cancer cell and tumor microenvironments. Wrong association or interpretation generates only misunderstanding and confusion.

As mentioned above, a same oncoprotein regulates different phenotypic traits of cancer cells. For example, it is frequently reported that inhibition of an oncoprotein in cancer cells leads to reduced tumorigenicity as shown by suppression of xenograft tumor formation in immunodeficient mice that lack T, NK and macrophage cells, and suppression of tumorigenesis. Detailed molecular mechanisms are usually found to be responsible for such an effect. In separate studies, inhibition of the same oncoprotein in cancer cells causes enhanced immunogenicity or sensitivity to antitumor immunity as shown by suppression of tumor formation in syngeneic mice with functional immune cells, and thus suppression of tumorigenesis. The effect is also interpreted as the result of elegant but different molecular signaling cascades. Typically, blocking an oncoprotein de-represses a particular immune related gene that is involved in regulation of immune cell activity or infiltration in a tumor. If putting these studies side by side, it is interesting to find that blocking an oncoprotein suppresses tumorigenesis no matter whether functional immune cells are present, raising a question what is the prime factor to consider, and whether it is appropriate to focus only on immunity when interpreting tumor suppression effect in response to blocking an oncoprotein. My recent results demonstrated that tumorigenicity and immunogenicity of cancer cells are inversely correlated with neural stemness. Induced differentiation of cancer cells causes loss of neural stemness and tumorigenicity, but enhancement of immunogenicity (Liu et al., 2025). The studies on differentiation of cancer cells into antigen-presenting cells analyzed their ability to promote anti-cancer immunity and underlying mechanisms in the context of immunity, but leaving tumorigenicity untested (Ascic et al., 2024; Linde et al., 2023). However, it can be predicted that cancer cells after differentiation into immune cells will inevitably lose their tumorigenicity even in an environment of severely impaired immunity, as shown by another study (Zimmermannova et al., 2023). This effect should not be interpreted purely as a consequence of anti-tumor immunity.

Quite many theories/hypothesis have been proposed to explain cancer, including the most widely recognized somatic mutation theory, the ecosystem theory, or the “tissue organization field theory (TOFT)” (Bhattacharya et al., 2025; Huang et al., 2025). Somatic mutations are among the many factors that drive cancer initiation. There are also many cancers contain no consistent driver mutations, and tissues free of cancer are found to harbor oncogenic mutations (Huang et al., 2025). Meanwhile, the mechanisms governing cancer metastasis cannot be explained smoothly by mutations only. The importance of somatic mutation can be interpreted in the following way. Cancer is a disease of cellular property change: loss of original cell identity and acquirement of neural stemness. Therefore, whether a mutation (or generally, any intra-/extracellular insults a cell suffers) drives cancer depends on whether it can confer to or promote neural stemness in cells. This is highly cellular context dependent. A mutation driving cancer in one type of cells does not necessarily mean that it drives cancer in another because of the difference in intra-/extracellular regulatory signals. The ecosystem theory tries to interpret multiple cell populations, their complex interactions, and populations’ evolvability by analogy with an ecosystem using ecological terminology (Bhattacharya et al., 2025; Aguadé-Gorgorió et al., 2024). The complex TME is indeed similar to an ecosystem to some extent. However, it seems that this analogy makes cancer TME, including concrete cell types, their interactions and evolving cell phenotypes, more abstract to understand. In a broader sense, any complex system can be considered as an ecosystem. Growth and differentiation of an embryo into an animal/human is also a good example of evolving ecosystem. As discussed earlier, the principle of neural induction during embryogenesis, which leads to generation of various types of cells/tissues, is also applicable to tumorigenesis. This might be helpful for understanding cancer ecosystem. TOFT was proposed to substitute for the somatic mutation theory. It emphasizes that 1) cancer is the disease resulting from tissue organization comparable to organogenesis, but not the disease of the cell, 2) proliferation is the default state of all cells, and 3) whether a cell becomes cancerous depends on the healthiness of the stroma of tissues/organs (Huang et al., 2025; Soto and Sonnenschein, 2005). But what tissue organization means remains to be clearly defined. Organogenesis of different organs is different processes and follows distinct hierarchy of differentiation and morphogenesis. This raises the question whether tumorigenesis resembles the organogenesis of the tissue/organ from which cancer is initiated. So far, there is no evidence at all to support the idea. By contrast, cancers with distinct origin are similar in molecular heterogeneity and lineage trajectories (Finlay et al., 2024). As tumor progresses, tissues gradually lose tissue-specific healthy organizations and acquire a convergent cancerous ecosystem (Shi et al., 2025). Additionally, there is also no evidence at all to support proliferation as the default state of all cells. Although TOFT was proposed to explain cancer as “development gone awry” (Soto and Sonnenschein, 2005), it does not involve any principles in developmental biology. Similar to “TOFT,” tumors are also proposed to be “outlaw organs” comprising multiple cell types (Hanahan, 2026). But how “an outlaw organ” looks like is not defined at all. It is also not clarified, for example, whether hepatocellular carcinoma is outlaw liver or outlaw breast or both, or what type(s) of outlaw organ(s) the teratocarcinomas or extraskeletal osteosarcomas look like. No evidence and scientific logic can be found to support such a proposal. It is rather questionable how a “concept” without minimal clarity can be helpful for understanding cancer. Besides these concepts, some pathological/physiological processes, such as inflammation, aging, have been proposed explain cancer. The effectiveness of a theory/hypothesis does not depend on the similarity in some particular details, which are convenient to find because of the systemic complexity of cancer, but depend on the following considerations: 1) whether it can clearly define cancer cell and cancer; 2) whether it can be experimentally verified; 3) whether it can explain cancer complexity as a whole, or integrate different aspects rather than specific details of cancer initiation and progression, and explain even the inverse correlation between cancer and neurodegeneration; 4) whether it can be translated into cancer therapeutic strategies.

## 7 Neural stemness being the core property of cancer cell paves the road to differentiation therapy of cancer

Differentiation therapy was suggested 50 years ago (Pierce and Wallace, 1971). Neural growth factors, all trans retinoic acid, arsenic trioxide, butyric acid or cAMP, showed some degree of differentiation-inducing capability in cancer cells. But differentiation therapy has been not applied as widely as other therapies. The best-known case of differentiation therapy might be the treatment of acute promyelocytic leukemia with all-trans retinoic acid (de Thé, 2018). The major obstacle should be the inappropriate understanding of the key property of cancer cells despite enormous studies. The core property of cancer cells being neural stemness, which endows cells with pluripotency, provides a general framework for differentiation therapy of different types of cancers: cancer cells can be induced to differentiate into different cell types by differentiation factors, particularly those driving embryonic tissue differentiation. A series of studies mainly in the 1970-80s demonstrated differentiation of cancer cells into benign cells within embryonic environment (Brinster, 1974; Cooper and Pinkus, 1977; Gerschenson et al., 1986; Gootwine et al., 1982; Hendrix et al., 2007; Illmensee and Mintz, 1976; Kulesa et al., 2006; Papaioannou et al., 1975; Podesta et al., 1984; Webb et al., 1984; Wells and Miotto, 1986; Mi et al., 1975). Unfortunately, these results did not get more attention later. Our studies have demonstrated that cancer cells can be induced to differentiate into neuronal-like cells by blocking endogenous oncoproteins, leading to loss of neural stemness and tumorigenicity in cancer cells (Zhang et al., 2022; Chen et al., 2021; Zhang et al., 2017; Lei et al., 2019). Embryonic non-neural lineage differentiation factors, GATA3, HNF4A, HHEX and FOXA3, were shown to suppress tumorigenicity via inhibition of neural stemness of cancer cells and suppress tumorigenesis in a mouse model of colorectal cancer (Yang et al., 2021). Immune cells are the key player in anti-cancer immunity. Differentiation of cancer cells into cells resembling immune cells was predicted based on cancer cell pluripotency (Cao, 2023). Indeed, *in vitro* and *in vivo* experimental data verified that cancer cells can be reprogrammed into antigen-presenting cells by lineage-specific specification factors. The resulting cells efficiently present tumor antigens and elicit systemic and safe antitumor immunity (Ascic et al., 2024; Linde et al., 2023; Zimmermannova et al., 2023). Colorectal cancer cells can be induced to differentiate into normal-like enterocytes, thereby suppressing malignancy (Gong et al., 2025). Moreover, tumorigenicity and immune privilege of cancer cells are coupled together by neural stemness. Induced differentiation, either by forced expression of lineage differentiation factors or by blocking endogenous oncoproteins, leads to loss of neural stemness, reprograms cancer cells from a tumorigenic and immune privileged state into a non-tumorigenic and immunogenic state, and reprograms the regulatory networks of cancer cells into those of differentiated cells (Liu et al., 2025). Most cancer promoting proteins are neural stemness factors, targeted therapies via inhibition of cancer promoting factors are in essence differentiation therapies. The efficiency relies on the uniformity of presence of a target in cancer cells and the degree of differentiation of cancer cells in response to target inhibition. In addition, cancer with inhibition of a target gene/protein might be more prone to signal feedback loops in cancer regulatory networks, which will ultimately lead to therapy resistance. By contrast, targeting neural stemness via induced differentiation driven by embryonic non-neural differentiation factors does not depend on a specific target molecule, can achieve more efficient differentiation effect, and reprogram overall cellular properties and regulatory networks of cancer cells. Such a strategy might avoid or alleviate therapy resistance effect elicited from complex signal feedback loops. I hope this review may attract more attention from researchers such that further efforts could be invested to explore the clinical application of differentiation therapy via targeting neural stemness.

## 8 Conclusion

Cancer is a systemic disease. Understanding a systemic disease needs the understanding of systemic rules. In my opinion, cancer is not solely the problem of mutations, not solely the problem of change in single genes or proteins, not solely the problem of metabolism or immunoevasion, and so on. But rather, cancer is the manifestation of the power of the rules dictating the animal kingdom: 1) The core property of cancer (tumorigenic) cell is neural stemness, which represents general stemness. Neural stemness unifies phenotypic traits of cancer cell, such as proliferation, matastasis, stemness, immune privilege; 2) Evolutionarily predetermined advantage of neural genes and unicellular origin of neural stemness determines and unifies pluripotent differentiation potential and tumorigenicity; 3) Pluripotency and tumorigenicity are both but different manifestations of the same cellular property, the neural stemness, during embryonic and postnatal stages of animal life, respectively; 4) Tumorigenicity is by nature the manifestation of aberrant occurrence of pluripotent state or neural stemness in a postnatal animal/human; 5) Tumorigenesis represents a process of progressive loss of original cell identity and acquirement of neural stemness; 6) Neural induction drives body axis formation during embryogenesis (and ectopic neural induction causes a conjoined twin), whereas a neural induction-like process drives tumorigenesis in postnatal animals/humans. Therefore, tumors are degenerated forms of conjoined twin structures; 7) Induced differentiation of cancer cells by embryonic differentiation factors reprograms cellular property and regulatory network of cancer cells, leading to loss of neural stemness, tumorigenicity, and differentiation potential, and enhancement of sensitivity to anti-tumor immunity. I suggest understanding cancer as a systemic disease by understanding neural stemness as the core property of cancer cells, the general rules dictated by neural stemness, and the principle of embryonic differentiation, and suggest developing cancer therapy by targeting neural stemness via efficient differentiation.
